# Impact of Role Conflict on Intention to Leave Job With the Moderating Role of Job Embeddedness in Banking Sector Employees

**DOI:** 10.3389/fpsyg.2021.719449

**Published:** 2021-11-19

**Authors:** Fazal Hussain Awan, Liu Dunnan, Khalid Jamil, Rana Faizan Gul, Aliya Anwar, Muhammad Idrees, Qin Guangyu

**Affiliations:** ^1^School of Economics and Management, North China Electric Power University, Beijing, China; ^2^Beijing Key Laboratory of New Energy and Low-Carbon Development, North China Electric Power University, Beijing, China; ^3^Department of Sociology, Government College University Faisalabad, Faisalabad, Pakistan

**Keywords:** role conflict, intention to leave, job embeddedness, Pakistan, bank employees

## Abstract

This study investigates why some employees intend to leave their jobs when facing conflict between family responsibilities and job routines. The present study also reveals the moderating role of on-the-job embeddedness between role conflict and intention to leave the job. Drawing on conservation of resources theory, the paper investigates the buffering effect of the three on-the-job embeddedness components (fit, links, and sacrifice). Data were collected from banking officers because most of the employees have to face role conflict between family and job responsibilities, as banking is considered among the most stressful jobs. Collected data were analyzed by applying structural equation modeling. Results indicate that the role conflict significantly influences intention to leave the job. Furthermore, the study shows that on-the-job embeddedness moderates the relationship between role conflict and intention to leave. The results suggest that organizations can reduce turnover intention during times of work and life conflict by developing employee on-the-job embeddedness. This study provides some insights to managers on why many employees leave their jobs and how to overcome this problem. Management should also offer extra and available resources in periods of greater tension to minimize early thinking regarding quitting.

## Introduction

Employee turnover is not a new trend. Millions of people around the world leave their jobs annually. Despite all the public benefits that are attached to being employed, employees decide to quit. The researchers found several variables that affect the choice to leave during periods of work-life imbalance (Aboobaker et al., 2017). Existing literature draws our attention to multiple factors which lead to the Intention of an employee to leave and employee turnover. Those factors include internal factors related to the job itself and external factors associated with a person’s personal and social life (Mansour and Tremblay, 2018). Numerous investigators have found that certain organizational factors, including organizational commitment, organizational support, and supervisors’ support, along with some social and personal stress factors, might trigger Intention to leave a job among employees (Zhang et al., 2019). Role conflict is one of those factors which might lead employees toward job quitting. Role conflict refers to one’s unpleasant experience, which one faces due to the confronting and clashing demands of different social roles and statuses (Anand and Vohra, 2020). Two types of role conflicts include intra-role conflicts and inter-role conflicts. Intra-role conflict refers to the conflict a person faces due to increased mental pressure and stress due to conflicting expectations and demands from one domain of life. For example, the tension in the job due to multiple responsibilities can be considered a result of the intra-role conflict. Another type of role conflict is an inter-role conflict that a person faces due to conflicting demands from different life domains (Mansour and Tremblay, 2016). For example, an employee’s inability to be a good parent or partner due to a demanding professional life can be considered the inter-role conflict that might lead them to quit their job (Dai et al., 2019).

When an employee leaves a job, they have to face two possible consequences. The person who leaves a job either joins another organization or is left unemployed (Lee et al., 2020). In the case of unemployment, a person faces several consequences. The financial situation of the quitter is an immediate and distressing outcome of sudden unemployment (Bayarcelik and Findikli, 2016). It is well documented that not every person gets a new job right after leaving an employer. Even for a few months, a break in the regular inflow of income can put the person into further distress (Samadi et al., 2020). Financial instability then leads to different issues related to a person’s health, mainly psychological conditions. Unemployed people commonly report depression and anxiety for a long time (Minamizono et al., 2019). Social outcomes emerge in the shape of worry about dependents’ financial security and the form of social gatherings being restricted (Jyoti and Rani, 2019).

Every organization aims to have high productivity and consistently increasing profitability. High profits are indicators of the success of companies. Employees are significant contributors to a company’s success (Gyensare et al., 2016). The consequences of employee turnover for the employer are frequently documented. An employee’s sudden departure can hinder the operations of an office for some time (Sun and Wang, 2017). It also affects team dynamics within an organization. Organizations invest a lot in their employees’ training (Cho et al., 2017). All the company’s investment in an employee is lost when a trained employee no longer continues to work in the organization (Lee, 2018). Highly motivated individuals serve as the driving forces of profit and competitiveness (Zimmerman et al., 2019). Along with the increase in productivity and profits, organizations try to have lower turnover as the managers are familiar with the importance of employee retention (Stamolampros et al., 2019). Numerous researchers have stressed the importance of employee retention and recommended incorporating retention strategies as fundamental principles in organizational policies (Degbey et al., 2021). Adoption of retention strategies is also recommended to small and medium sized business enterprises for the achievement of consistent productivity and rising profits (Alhmoud and Rjoub, 2019).

This research investigates if on-the-job embeddedness affects the connection between work-life conflict and an employee’s intention to leave. Job embeddedness theory describes an employee’s retention due to an employee’s unique set of ties to the organization. In addition, it has been shown that an employee’s embeddedness may serve as a buffer against bad events and ill circumstances. This study contributes to the work-life balance and job embeddedness literature by investigating whether on-the-job embeddedness moderates the effect of work-family conflict on employee leaving intention.

On the other hand, numerous attempts have been made to understand the association between role conflict and Intention to leave. Still, no investigations have been made so far which are specifically directed toward the banking sector. When it comes to the context of Pakistan, the literature seems even more limited on the subject. Therefore, this investigation’s purpose is the empirical explanation of the association between role conflict and Intention to leave and the role of job embeddedness as a mediator in their relationship in the banking sector of Pakistan. This study will contribute to the existing literature by producing information about the causal relationship among role conflict, Intention to leave, and job embeddedness. This study is also aimed to provide an empirical understanding of all these concepts in the context of Pakistan’s Banking sector for the development of effective retention policies by the banks.

The following paragraphs are a brief review of relevant literature and are followed up by the hypothesized relationships between focal constructs. Then the research methods, analyses, and findings are recorded and discussed. Finally, both the theoretical and managerial implications and the limitations and avenues for future research have been discussed.

## Review of Literature

A review of the literature about the relevant concepts and sectors is narrated below.

### Intention to Leave

Sasso et al. (2019) revealed that Intention to leave or intention to quit refers to the potential plans to leave the job with an employer. It is a global phenomenon. Multiple factors include personal factors and employers’ behavior, performance appraisal and feedback, absenteeism, burnout, lack of recognition, personal and professional advancement, miscommunication, and job satisfaction (Holland et al., 2019). A literature review of some of the critical factors that have been reported to be associated with leaving is presented in the following paragraphs.

Employers’ attitude has been reported frequently in the literature as an indicator that triggers the intention to leave in an employee (Meriläinen et al., 2019). The majority of the people who leave their job have mentioned the employers’ unprofessional attitude as the main factor that led them to quit. Numerous researchers have reported that employees are less likely to quit their jobs if they think their employers are professional (Chin et al., 2019). The second important factor that has been frequently used in the literature is appreciation and performance appraisal. Several researchers have narrated that workers feel motivated when their managers and colleagues appreciate their work (Lo et al., 2018). Positive feedback on one’s work is associated with improved performance. Employees have reported in numerous studies that appreciation inspires them to work better and harder with increased interest. However, investigators have noted that many organizations lack effective performance appraisal mechanisms (Yamaguchi et al., 2016).

de Oliveira et al. (2017) discussed that an ineffective appraisal system gives rise to a lack of recognition. Employees have reported that they work very hard to get credit for their contribution to an enterprise. As a result, they are left with a sense of worthlessness. This directly demotivates the workers and causes lessening interest in their work (Mihail, 2017). This affects the productivity of a firm negatively. Workers who left their jobs have informed us in the surveys that they were not concerned with the firm’s overall productivity due to lack of recognition of their efforts at the workplace. People do not give their best at work when their work is not credited (Bohle et al., 2017).

Mahomed and Rothmann (2020) expressed that leaving is damaging for both employees and employers. An employer’s investment in a managerial level employee’s training is wasted if they quits the job (Asakura et al., 2020). A new employee takes a lot of time to understand the organizational environment and working mechanism. An organization has to invest in a new employee’s training when an experienced manager leaves the organization (Domínguez Aguirre, 2019). If the intention to leave leads to job quitting, it can create personal, social, and psychological problems for the quitter (Loi et al., 2006).

Not every firm understands the importance of employee retention, as it has been reported that not all firms adopt retention strategies (MacIntosh and Doherty, 2010). However, many firms adopt different approaches and techniques to retain their workers. Retention strategies vary from firm to firm (Frye et al., 2020). Incentives, increments, competitive remuneration packages, promotions, appraisal systems, training, and recognition of work along an attractive working environment are among the tools commonly used by employers for employee retention (Silva et al., 2019).

### Role Conflict

The concept of “role conflict” defines a kind of internal conflict in which job and family role obligations are at odds with one another to some degree, making it difficult to fulfill requirements in one area while having to meet them in the other (Zainal Badri and Wan Mohd Yunus, 2021). Both work and family roles may be defined by the obligations placed on the individual by their work colleagues and family members and the values the person has about their work and family role behavior. When work and family needs clash, it is easy to believe that the workplace is interfering with family happiness and pleasure (Díaz-Fúnez et al., 2021).

Role conflict may also arise when family issues interfere with job happiness and job performance. Dividing time between job and family (multiple roles) may lead to inter-role conflict since these responsibilities may deplete one-another’s resources (Del Pino et al., 2021). To fulfill the needs of one job, the expectations of the other roles are ignored. If family responsibilities come after the job, the family strain may detract from the ability to fulfill the job function (Gul et al., 2021).

Inter-role conflict and intra-role conflict are two commonly used concepts in the literature of role conflict. An inter-role conflict is a form of role conflict that refers to stressful conditions resulting from conflicting demands from different life spheres (Iannucci and MacPhail, 2018). Workers have frequently reported the inter-role conflict due to continuous pressure from the employer and the increasing demands of family members (Karim, 2017; Sarfraz et al., 2021). Intra-role conflict, another form of role conflict, is associated with a single role’s expectations and needs. It can occur due to either expectations in the workplace or family members’ demands (Grzywacz, 2020). The employees frequently report Intra-role conflict. Both inter-role conflict and intra-role conflict cause employees to experience role strain. Role strain is another often-used concept in the literature regarding role conflict (Wendling et al., 2018). Role strain can be explained as tension that a person experiences when he/she faces competing demands within one particular role and find it challenging to perform according to the expected roles (Jamil et al., 2021).

Inter-role conflict can occur due to multiple reasons, but role ambiguity is reported as a common reason people experience role conflict at the workplace (Wehner, 2016). Role ambiguity is when an employee lacks awareness about their job-related duties (Oviatt et al., 2017). Numerous researches demonstrate that employees who do not perform as per employers’ expectations are usually unclear about their responsibilities in the workplace (Purohit and Vasava, 2017). That role ambiguity is reported mainly as a result of miscommunication. Role ambiguity can be overcome through clear communication of job responsibilities and duties (Furtado et al., 2016).

Role conflict may also result from workers’ failure to manage their work and family (non-work) obligations on an equal footing (Yousaf et al., 2020). This kind of conflict may suggest that employees’ job duties interfere with their happiness and success in their personal lives or that employees’ personal lives interfere with their satisfaction and success at work (Naseem et al., 2020). Therefore, it is probable that role conflict will have adverse effects, such as stress and dissatisfaction, and interfere with the ability to fulfill work or family obligations (Baranik and Eby, 2016; Mohsin et al., 2021). Furthermore, this tension may result in voluntary turnover, according to studies conducted by Eby et al. (2014), which supports this assertion. Hence, we proposed the following hypothesis:

H1: Role Conflict has a significant impact on intention to leave a job.

### The Moderating Role of Job Embeddedness

The term “on-the-job embeddedness” refers to a worker’s connection to social ties formed at work, making them reluctant to quit the company (Jiang et al., 2012). A detailed explanation is given by Lee et al. (2004) about the effect of on- and off-the-job embeddedness on job performance, attitude related to firm citizenship, and also decrease and lower the impact of truancy on global turnover (Amjad et al., 2018). Sekiguchi et al. (2008) demonstrated that there is a strong bond between leadership-membership exchange (LMX) and task action, LMX and organizational citizenship behaviors (OCBs), organization-based self-appreciation, and is reinforced by job embeddedness factor (which is the composition of on-the-job and off-the-job embeddedness). Karatepe (2012) has depicted that embeddedness reinforces the negative effect of well-known firm and co-worker support (Karatepe, 2012; Naseem et al., 2021) and also increments the impacts of appreciation of organizational and firm equity (Karatepe and Shahriari, 2014) on representative leaving out proposals (Dunnan et al., 2020a). A demonstration was given by Özçelik and Cenkci (2014) about employee embeddedness that a worker with a fundamental level of work embeddedness detailed a weaker interconnection between finding work and turnover than those workers who have lower levels of embeddedness. Burton et al. (2010) demonstrated some interconnection between paternalistic administration and work in-role execution. Peachey et al. (2014) have detailed research regarding embeddedness and have shown that the impact of unfurling theory-type stuns on worker progress can be weakened by embeddedness. It fortifies the effect on OCB. Peachey et al. (2014) demonstrated that employee embeddedness strengthened the connection between workers’ past encounters of work environment bullying and consequent work environment hostility. Al-Ghazali (2020) explained that worker embeddedness could fortify the effect of worker recognitions of procedural decency and transmission on danger evaluation and organizational rebuilding.

#### The Moderating Effect of On-the-Job Fit Embeddedness

This study claims that on-the-job fit embeddedness is a valuable resource for helping employees deal with work-life balance issues (Kiazad et al., 2014a). Work-life conflict is less likely to impact workers with greater fit embeddedness. Consequently, there is a minor link between work-family conflict and intention to leave and companies with lower fitness levels. Two mechanisms have been suggested:

First, those employees with a better person-job fit are more likely to have more essential work skills, making it easier for them to fulfill the ordinary demands of their position compared to employees with a worse fit (Dunnan et al., 2020b). Second, employees with low levels of person-job fit will deplete resources more quickly to fulfill the day-to-day work needs than those with high levels of person-job fit. Third, the extra depletion of resources that results from role conflict between the job and home domains adds to the stress and exhaustion felt in both fields (Al-Ghazali, 2020). As a result, workers who aren’t a good match for the company will be hurt more. As a result, workers who fit in well on the job are less likely to be impacted and less likely to be motivated to leave.

Second, workers who better match the work-family conflict needs of the workplace may potentially adapt. Fasbender et al. (2019) suggest that conflict between a person’s ideas and expectations and the reality they encounter prompts an employee to change their stance and attitudes. People that are more in sync are better able to find and create new workplace arrangements (Chan et al., 2019; Naseem et al., 2020b). Employees with better degrees of fit embeddedness will feel less of a negative impact as a result. Hence, we proposed the following hypothesis:

H2: On-the-job fit positively moderates the relationship of role conflict and intent leave job.

#### The Moderating Effect of On-the-Job Link Embeddedness

Employees who have a greater level of on-the-job link embeddedness are more likely to know more individuals at the organization and be more engaged in the organization’s matters than their counterparts (Lyu and Zhu, 2019). Because of this connectivity, workers have greater access to possibilities inside the organization and services like career sponsorship from higher-ranking colleagues (Coetzer et al., 2018). Thus, link embeddedness may be seen as a valuable resource that allows employees to more easily access ameliorating support services offered by the organization in which they work. This has the potential to operate in two ways. First, increasing levels of social connection throughout an organization may, in the first instance, offer personal support for those who are experiencing problems as a result of work-family conflict (Sender et al., 2018). Second, employees who have built up social capital can better identify and negotiate administrative support services, such as organizational work-life balance programs, feel more comfortable accessing these services, and obtaining supervisor support. In a recent study, researchers found that some employees are hesitant to use organizational support services and that employee use of family friendly corporate resources can be influenced by the quality of employee-manager relations (Jamil et al., 2021b). Workers with a greater degree of link embeddedness had an easier time implementing current ameliorative measures, resulting in a reduction in the experience of stress resulting from work and family conflict compared to employees with a lower level of link embeddedness. Hence, we proposed the following hypothesis:

H3: On-the-job link positively moderates the relationship of role conflict and intent leave job.

#### The Moderating Effect of On-the-Job Sacrifice Embeddedness

While fit and connection embeddedness has been suggested to mitigate the impact of work-life conflict on leaving intention, sacrifice embeddedness has been proposed to enhance that relationship, following Zhang et al., (2019). According to COR theory, workers with a higher level of sacrifice embeddedness are more likely to accumulate intrinsic resources than employees with a lower level of sacrifice embeddedness. When intrinsic resources are collected in larger quantities, there is a greater likelihood of eventual loss. At the same time, unlike instrumental resources, increases in intrinsic resource levels do not always result in an improvement in an entity’s ability either to withstand threats or obtain additional resources (Hobfoll, 2011).

According to COR theory, a threat to current resources will elicit a more significant response than an opportunity to acquire new resources or invest existing resources in the short term (Hobfoll, 2011). Employees with more to lose, such as high levels of intrinsic resources, will be more sensitive to risks, such as the depletion potential of work and life conflict since they have more to lose. Kiazad et al. (2014b) discovered that when confronted with a threat such as a psychological contract breach, employees with higher sacrifice embeddedness reacted more strongly in defense of their existing resources when compared to employees with lower sacrifice embeddedness (Kiazad et al., 2014). Those workers who had a high level of sacrifice embeddedness used a resource defense strategy to reduce their exposure to the resource-depleting danger, which was shown to be effective in that research.

**FIGURE 1:**
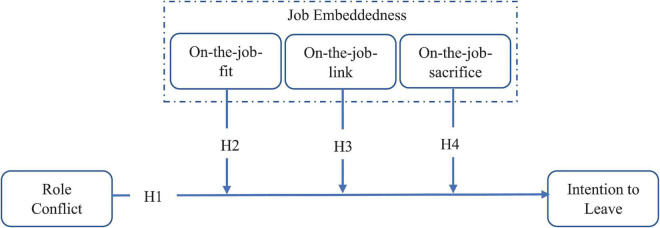
Conceptual framework.

According to the findings of this research, workers who have a higher level of sacrifice embeddedness are more likely to undertake resource-saving measures, which may include considering quitting the organization (Stewart and Wiener, 2021). As a result of being unable to ward off threats to resources or obtain replacement resources, workers who have made significant sacrifices are more inclined than employees who have less to lose than they are to contemplate quitting the organization to protect their existing resources (Amoah et al., 2021). Therefore, workers with greater degrees of sacrifice embeddedness are more likely to experience conflict at work and home, and they are also more likely to consider quitting their current position. Hence, we proposed the following hypothesis:

H4: On-the-job sacrifice positively moderates the relationship of role conflict and intent leave job (see Figure 1 for all relationships).

## Methodology

### Data Collection and Sample Size

The study samples include branch managers, operations managers, credit officers, and all officers, including grade one, two, and three major private banks situated in three big cities of Pakistan: Faisalabad, Lahore, and Islamabad. Also, we only included respondents who had been working in the banking sector for more than 5 years. The average experience of respondents in the banking sector was 8.5 years, and their time at their current location was 2.5 years. A pilot study with 30 participants was carried out. Since providing recommendations, revisions were made to the final questionnaire to make it more understandable for respondents. To ensure the content validity of the measures, three academic experts of human resource management analyzed and made improvements in the items of constructs. The experts searched for spelling errors, grammatical errors, and ensured that things were correct. The experts have proposed minor text revisions to role conflict and job sacrifice items and advised that the original number of items be maintained. The sample size was determined by using Kline (2015) proposed criterion. He suggested at least ten responses per item. Therefore, a minimum of 180 samples was needed, given the 18 items in this study. To increase reliability and validity, 250 questionnaires were distributed to research participants. At the time of scrutiny, 30 questionnaires were found incomplete, and these questionnaires were excluded, and the final sample is 220 respondents.

### Questionnaire and Measurements

The study used items established from prior research to confirm the reliability and validity of the measures. All items are evaluated through five-point Likert-type scales where “1” (strongly disagree), “3” (neutral), and “5” (strongly agree).

Role Conflict was measured with five items adapted from the study of Netemeyer et al. (1996); the sample item is, “The demands of my work interfere with my home and family life.”

Job Embeddedness, with its three dimensions: on-the-job fit, on-the-job link, and on-the-job sacrifice, was assessed with items adapted from the study (Felps et al., 2009). Job Embeddedness was measured with nine items; on-the-job fit consisted of three items, and the sample item is, “I feel like I am a good match for my organization.” The on-the-job link consisted of three items, and the sample item is, “I work closely with my coworkers.” Finally, on-the-job sacrifice consisted of three items, and the sample item is, “I would sacrifice a lot if I left this job.”

To assess the intention to leave, the four items were adopted from the work of Abrams et al. (1998) with the sample item, “In the next few years, I intend to leave this company.”

#### Demographic Characteristics

This study analyzed the data through Smart partial least squares (PLS), primary data was collected from 220 respondents, demographic characteristics of respondents such as age, gender, education, income, and experience are illustrated in Table 1.

**TABLE 1:** Demographic characteristics of respondents.

Categories	Subcategories	Size	Percentage
Gender	Male	150	68
	Female	70	32
Total		220	100
Age	20–30	40	18
	31–40	70	32
	41–50	80	36
	51 and above	30	14
Total		220	100
Family Status	Nuclear	100	45
	Joint	70	32
	Extended	50	23
Total		220	100

## Data Analysis

The study used PLS modeling using Smart-PLS 3.2.8 version (Ringle et al., 2015) as the numerical tool to analyze the structural and measurement model, as it can accommodate a smaller number of observations without normality assumptions and survey research is generally not normally distributed (Chin et al., 2003). Also, since data were collected using a single source, we followed Kock (2015) to test the common method bias using the full collinearity method. The test showed that all the VIFs were lower than five; thus, we can conclude common method bias is not a severe problem in our study (Sánchez-Hernández et al., 2020).

### Reliability and Validity of the Constructs

**FIGURE 2:**
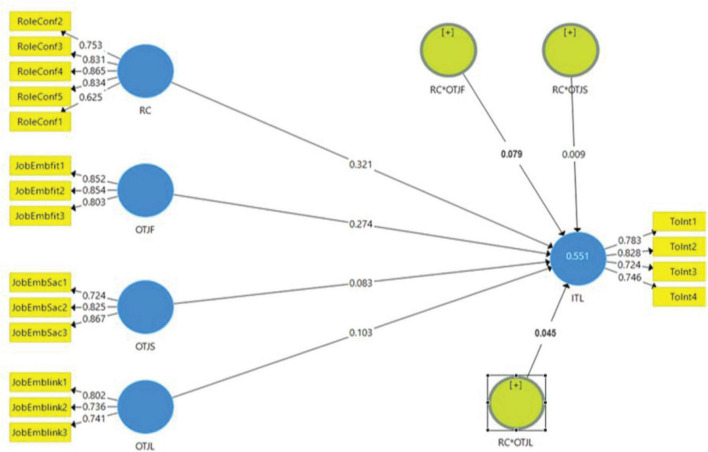
Measurement model.

The Measurement model explains the relationships among the constructs and the indicator variables. As part of the measurement model assessment, all the indicators held factor loading greater than 0.60 and were retained in the model (Gefen and Straub, 2005). The reliability analysis is the first element of the measurement model, which contains composite reliability. According to Ramayah et al. (2018), the composite reliability’s required threshold value is 0.70. Subsequently, the indicators’ findings are greater than 0.7, confirming the measurement model’s composite reliability (see Table 2). The composite reliability values of all the constructs are also greater than 0.7, which further strengthens the reliability of all the variables (see Figure 2). Convergent Validity evaluates whether or not constructs measure what they are supposed to measure. In this study, convergent Validity was assessed by calculating the average-variance-extracted (AVE) that shows whether the construct variance can be described from the selected items (Fornell and Larcker, 1981a). According to Bagozzi and Yi (1988), the cut-off value for the average variance extracted is 0.5, and the Values of AVE of all constructs are greater than the recommended threshold, as shown in Table 2. This reflects the convergent Validity of the measurement model. Table 3 displays the discriminant validity assessment whereby the HTMT ratios were all below the 0.90 cut-off value. The confidence intervals do not include a zero or one, as suggested by Henseler et al. (2016). Thus, we can conclude that the measures used in this are reliable, valid, and distinct.

**TABLE 2:** Reliability, validity, and descriptive of the measures.

Constructs	Items	Items Loading	Skewness	Kurtosis	Alpha	CR	AVE	SE_skew	SE_Kurt
Intention to Leave	ITL1	0.783	−1.3766	2.4867	0.7749	0.8541	0.5946	0.164	0.3266
	ITL2	0.828							
	ITL3	0.724							
	ITL4	0.746							
On-the-job-fit	OTJF1	0.852	−1.0434	1.2538	0.7909	0.8746	0.6993	0.164	0.3266
	OTJF2	0.854							
	OTJF3	0.803							
On-the-job-link	OTJL1	0.802	−0.8708	1.3622	0.7784	0.8042	0.5783	0.164	0.3266
	OTJL2	0.736							
	OTJL3	0.741							
On-the-job-sacrifice	OTJS1	0.724	−1.0955	1.4048	0.7566	0.8483	0.6522	0.164	0.3266
	OTJS2	0.825							
	OTJS3	0.867							
Role Conflict	RC1	0.625	−1.6315	2.4645	0.8784	0.8889	0.6184	0.164	0.3266
	RC2	0.753							
	RC3	0.831							
	RC4	0.865							
	RC5	0.834							

**TABLE 3:** Discriminant validity (HTMT Ratios).

	ITL	OTJF	OTJL	OTJS_	RC
ITL					
OTJF	0.804				
OTJL	0.656	0.787			
OTJS_	0.632	0.711	0.877		
RC	0.696	0.582	0.369	0.449	

### Discriminant Validity

In short, the Fornell and Larcker technique demonstrates discriminant validity when the square root of the AVE enhances the relationships between the measure and every other measure. To stimulate the measurement of the model’s discriminant validity, the AVE estimation of every construct is produced using the Smart-PLS algorithm, as shown in Table 3.

The values that lie in the off-diagonal are smaller than the average variance’s square root (highlighted on the diagonal), supporting the scales’ satisfactory discriminant validity. Consequently, the outcome affirmed that the Fornell and Larcker (1981b) model is met.

### Structural Model

**FIGURE 3:**
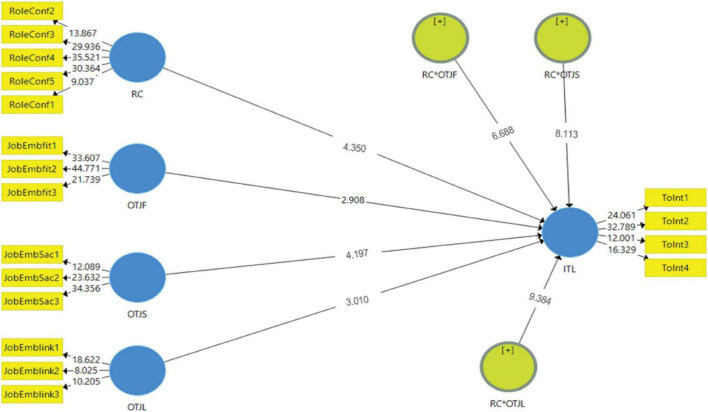
Structural model.

The structural model reflects the paths hypothesized in the research model and is assessed based on multicollinearity, coefficient of determination *R*^2^, predictive relevance *Q*^2^, and the paths’ significance (see Figure 3). The goodness of the model is determined by the structural path’s strength, determined by the *R*^2^ value for the dependent variable (Hair et al., 2014). All the VIFs were below five; thus, this confirms that the structural model results are not negatively affected by collinearity. Furthermore, following the thumb rules, the *R*^2^ values of intention to leave (0.551) exceed the minimum value of 0.1 suggested by Falk and Miller (1992), confirming a satisfactory predictability level. Furthermore, the *Q*^2^ value of the endogenous construct is considerably above zero, thus providing support for the model’s predictive relevance regarding the endogenous latent variables.

Next, to assess the four hypotheses developed we ran a bootstrapping of 5,000 subsamples. First, we assessed the direct relationships before looking at the moderation effects. The results revealed a significant relationship between role conflict and intention to leave (β = 0.32, *p* < 0.01, BCI LL = 0.169 and BCI UL = 0.463) which gives positive support for H1 of our study. The moderation hypotheses of the job embeddedness in the path between role conflict and intention to leave (H2, H3, and H4) are tested using the two-stage continuous moderation analysis (Hair et al., 2017). The moderating effect of RC × OTJF → ITL (β = 0.047, *p* < 0.01, BCI LL = −0.193 and BCI UL = 0.298), RC × OTJL → ITL (β = 0.089, *p* < 0.01, BCI LL = −0.417 and BCI UL = 0.061), and RC × OTJS → ITL (β = 0.023, *p* < 0.01, BCI LL = −0.115 and BCI UL = 00.22) indicating the moderating effect are statistically significant at the 0.01 level. This gives support for H2, H3, and H4 of this study (see Table 4).

**TABLE 4:** Hypotheses results.

Hypothesis	Relationships	Std. Beta	Std. Error	*t*-value	*p*-value	BCI LL	BCI UL
H1	RC → ITL	0.32	0.075	4.292	0.000	0.169	0.463
H2	RC × OTJF → ITL	0.047	0.096	6.688	0.000	−0.193	0.298
H3	RC × OTJL → ITL	0.089	0.077	11.385	0.000	−0.417	0.061
H4	RC × OTJS → ITL	0.023	0.083	8.113	0.010	−0.115	0.22
*Endogenous construct*		*R* ^2^	*Q* ^2^				
ITL		0.551	0.295				

**FIGURE 4:**
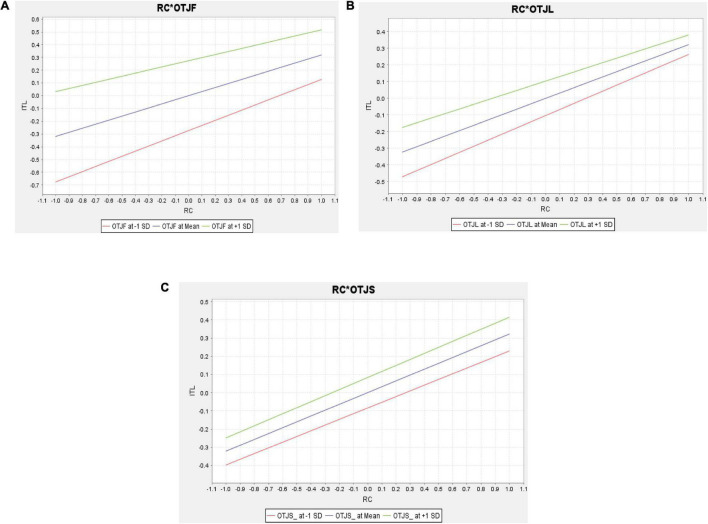
Moderating effects.

Also, for H2 the moderation graph indicates at the low level of On-the-Job Fit, there is a low impact of RC on ITL. However, increasing OTJF enhances the significant positive effect of RC on ITL (see Figure 4A). Moreover, the H3 moderation graph describes at the low level of the On-the-Job link there is a low impact of RC on ITL. Again, though, increasing OTJL augments the significant positive effect of RC on ITL (see Figure 4B). Finally, the final relationship graph shows at the low level of On the Job sacrifice, and there is a low impact of RC on ITL. Nevertheless, increasing OTJS improves the significant positive effect of RC on ITL (see Figure 4C).

## Discussion and Conclusion

This present research aims to determine whether job abandonment moderates the relation between role conflict and intention to leave in the banking sector. For highly embedded employees in the banking sector, role conflict is a common occurrence. This study analyses job embeddedness in terms of employee retention. In particular, the results suggest that job embeddedness moderates the relationship between the WFC and job turnover intention. The results indicate that role conflict and job embeddedness are opposing factors that lead people to quit or remain within their banks. The results suggest that job embeddedness connects workers within the organizations (Lee et al., 2004; Jiang et al., 2012) is supported by the theory of embeddedness (Mitchell and Lee, 2001). This means that job embedding has a beneficial influence when companies minimize “dysfunctional” revenue.

In terms of the suggested hypothesis, all three moderating effects are straightforward to comprehend. Employees who have a high level of linkage embeddedness have a bank of instrumental resources at their disposal that may help them cope more effectively with the difficulties of work and family conflict. The stronger the employee’s connection to the organization and the higher the linkage embeddedness, the better the employee can access and utilize a variety of existing organizational resources (Jung and Kim, 2021). Employees who are more integrated into their work will be more able to obtain inter-personal assistance from their colleagues and will be less reluctant and more effective in using existing organizational support systems due to their integration. So, employees reporting a lower connection between work and family conflict and leaving intention are more likely to be those who had more significant degrees of linkage embeddedness in their job.

Burton et al. (2010) showed that the embeddedness of workers reinforced the link between the history of bullying in a workplace and resulting violence in the workplace. Theory and studies on embedding work typically indicate that job embeddedness in the workplace has beneficial effects where the needs of workers and institutions are matched. In other terms, workers are trained to use working connections, health, and compromises to delegate services in the job domain and institutions to embedded employees. Because of the limitations generated by role conflict, our results indicate that an engaged employee with a high degree of embeddedness is more likely than a low embedded working employee to feel mental fatigue, remorse, and aggression. Thus, the high degree of job-embedded employees is more likely to be adversely impacted by role conflict. By concentrating on previously untested competing factors, this research leads to sparse work on the “dark side” of work integration (Sekiguchi et al., 2008; Burton et al., 2010; Allen et al., 2016).

This research provides deep insights into the moderating role of job embeddedness between role conflict and employees’ intention to leave and exposes significant shortcomings on the theory of work-building. This study also leads to limiting role conflict implementing and job embeddedness measures (Oviatt et al., 2017). In addition, the results indicate that those who are more deeply embedded in the job may improve their resource investment in the role conflict, as this relationship with turnover has been negatively impacted by a higher degree of job embeddedness. However, around the same period, these working parents became more vulnerable to mental fatigue, remorse, and hostilities because of resource drain. With these negative findings in mind and a high degree of embeddedness, this research goes beyond recent studies on the moderating impact of convergence between role conflict and turnover (Ringle et al., 2015).

## Theoretical and Practical Implications

The studies provide several implications. First of all, this paper shows that Job embeddedness has a moderating impact on the relationship between role conflict and turnover intention. This contributes to the role conflict literature by extending the literature of job embeddedness. Essentially, the three elements of job embeddedness do not function as a unifying entity in this paper. Instead, there were differing impacts to the three components on the job. The fitness of embedding had no effect; embedding the link had an improving outcome, and embedding sacrifices had a multiplier effect. This shows no established linkage between embedded components on the job (Kiazad et al., 2014; Bohle et al., 2017). JET researchers should therefore use the three on-the-job parts instead of combined measures such as job integration or work-based embedding. Second, this article shows the efficient justification of how job embedders moderates the impact of role conflict on the employee’s intention of turnover by COR theory. This research adds to an earlier study’s findings into role conflict, strain, and burnout that explains the impact of work and family conflict on the depletion and acquisition of employee resources (Ghosh et al., 2017).

In addition to that, this paper would discuss plans to establish processes to understand how work incorporation impacts perceptions and attitudes (Kiazad et al., 2014). Finally, in connection with this study, the increasing COR research describes the influence of employee embeddedness as a form of resource abundance (Kiazad et al., 2014; Wehner, 2016).

This study has three Practical Implications. Firstly, by improving the usage by workers of current changes in the company to minimize the impact of role conflict, the detrimental effects of role conflict will be minimized. This research indicates that workers with stronger linkage embeddedness are more capable of using interpersonal and organizational resources. The administration would enhance the efficiency of existing ameliorating arrangements to increase employee interaction, subordinates, and organization (Holtom, 2016). Finally, the design of corporate training programs, the growth relating to teamwork judgment, organizational training and professional development programs, social networks within the company, and employee recruiting schemes are used to improve employee engagement embedding (Treuren, 2019).

Secondly, introduce measures to make employees’ connections to coworkers, bosses, and the company more effective. To create a relationship between employees and the company, mentorship programs may be set up. In addition, teams can have work and decision-making responsibilities expanded, in-house training and career development programs implemented, and referral systems used to hire employees. The various programs may be tailored to the preferences and requirements of different groups of employees.

Thirdly, even though workers with greater sacrifice embeddedness had fewer intentions to leave their jobs, these individuals are more sensitive to work and family conflict than employees with lower levels of sacrifice embeddedness. Therefore, at times of more conflict, management may offer extra and readily available ameliorative services to reduce the likelihood of early departure thoughts.

## Limitations and Further Research

There are also limitations to this study. First, it employs cross-sectional measures and studies in social sciences to deduce the complexities of workers’ behavior. Longitudinal research is required to explain the effect and the purpose of job embedding on role conflict and turnover intention. Second, the research is focused on workers’ expectations and is thus susceptible to social desirability and the usual empirical biases. While the typical process bias in this study is minimal, more analysis could minimize the error of parameter estimation by having actual data on turnover or from various sources, for example, a supervisor or colleague, as the periods, have differed. Thirdly, the sample size is small (*n* = 220), which may have an issue of generalizability, leading to Type 2 errors and is also limited to the study. Finally, the limited size of the feminine cohort excluded substantial gender disparities from being investigated.

Further experiments should strive to obtain more significant balanced populations so that that gender effects can be compared. Furthermore, to determine the paper’s results, a single set of data from one sector of Pakistan was used; the findings are not necessarily generalizable. Uncertain jobs inside and outside the company may be calculated to be a biased estimation. The generalizability of these results would be explained further analysis into other geographic, social, and economic contexts. This paper outlines a structure for the influence of job embeddedness and role conflict and has defined new problems to address.

## Data Availability Statement

The datasets generated for this study are available on request to the corresponding author.

## Author Contributions

All authors listed have made a substantial, direct and intellectual contribution to the work, and approved it for publication.

## Conflict of Interest

The authors declare that the research was conducted in the absence of any commercial or financial relationships that could be construed as a potential conflict of interest.

## Publisher’s Note

All claims expressed in this article are solely those of the authors and do not necessarily represent those of their affiliated organizations, or those of the publisher, the editors and the reviewers. Any product that may be evaluated in this article, or claim that may be made by its manufacturer, is not guaranteed or endorsed by the publisher.
